# Female‐biased sex allocation and lack of inbreeding avoidance in *Cubitermes* termites

**DOI:** 10.1002/ece3.7462

**Published:** 2021-03-31

**Authors:** Veronica M. Sinotte, Benjamin H. Conlon, Elena Seibel, Jan W. Schwitalla, Z. Wilhelm de Beer, Michael Poulsen, Nick Bos

**Affiliations:** ^1^ Department of Biology Section for Ecology and Evolution University of Copenhagen Copenhagen East Denmark; ^2^ Leibniz Institute for Natural Product Research and Infection Biology Hans‐Knöll‐Institute Jena Germany; ^3^ Department of Microbiology and Plant Pathology Forestry and Agriculture Biotechnology Institute University of Pretoria Pretoria South Africa

**Keywords:** alates, kin recognition, mate choice, reproductive investment, sex ratio bias, *Wolbachia*

## Abstract

Sexually reproducing organisms face a strong selective pressure to find a mate and ensure reproduction. An important criterion during mate‐selection is to avoid closely related individuals and subsequent potential fitness costs of resulting inbred offspring. Inbreeding avoidance can be active through kin recognition during mate choice, or passive through differential male and female‐biased sex ratios, which effectively prevents sib‐mating. In addition, sex allocation, or the resources allotted to male and female offspring, can impact mating and reproductive success. Here, we investigate mate choice, sex ratios, and sex allocation in dispersing reproductives (alates) from colonies of the termite *Cubitermes tenuiceps*. Termites have a short time to select a mate for life, which should intensify any fitness consequences of inbreeding. However, alates did not actively avoid inbreeding through mate choice via kin recognition based on genetic or environmental cues. Furthermore, the majority of colonies exhibited a female‐biased sex ratio, and none exhibited a male‐bias, indicating that differential bias does not reduce inbreeding. Sex allocation was generally female‐biased, as females also were heavier, but the potential fitness effect of this costly strategy remains unclear. The bacterium *Wolbachia*, known in other insects to parasitically distort sex allocation toward females, was present within all alates. While *Wolbachia* is commonly associated with termites, parasitism has yet to be demonstrated, warranting further study of the nature of the symbiosis. Both the apparent lack of inbreeding avoidance and potential maladaptive sex allocation implies possible negative effects on mating and fitness.

## Graphical abstract

Cubitermes termites do not appear to avoid inbreeding through mate choice. Furthermore, investigated colonies generally had a female bias, and never a male bias, indicating the termites also do not avoid inbreeding through split sex ratios. Lastly, all investigated sexuals were infected with *Wolbachia*, which is transmitted only through females. This raises the possibility that this symbiont selfishly influences the termites' sex ratio.

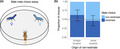

## 1 INTRODUCTION

For sexually reproducing organisms, selecting a mate and securing reproduction are critical steps in maximizing individual fitness. Mating with a relative can have high costs, including the expression of deleterious recessive mutations through increased homozygosity (Andersson & Hughes, 1996; Charlesworth & Willis, 2009; Pusey & Wolf, 1996). Inbreeding is avoided in many animals through kin recognition (Blouin & Blouin, 1988; Pusey & Wolf, 1996), sex‐biased dispersal (Noirot, 1989; Pusey & Wolf, 1996), and differential sex ratios, where individuals in a population specialize on producing predominantly males or females, consequentially reducing the likelihood of mating with a sibling (Vargo & Husseneder, 2010). Sex allocation, defined as the energy invested in male and female offspring, can influence the mating, resources procurement, and reproductive success of these individuals or their kin, ultimately impacting inclusive fitness (Fisher, 1930; West, 2009). For monogamous organisms, in particular, inbreeding or maladaptive sex allocation may carry substantial fitness costs.

The lifetime monogamy of termites means that their single mate choice has lasting consequences with regards to inbreeding. Unlike social Hymenoptera, termites are diplo‐diploid, and different selection pressures can affect whether inbreeding is avoided, preferred, or tolerated. Although inbreeding has been predicted to often be tolerated in many organisms due to increased fitness benefits of passing on related genes and increased parent‐offspring relatedness (Duthie & Reid, 2015), egalitarian parental care (such as in termites) is predicted to undermine this and lead to inbreeding avoidance (Kokko & Ots, 2006); for example through kin recognition or differential sex ratios. Furthermore, inbreeding should not be favored in monogamous organisms unless inbreeding avoidance carries a cost (Waser et al., 1986). Reproductive termites alates leave their natal nest in a nuptial flight, during which they must rapidly choose a mate for life amidst substantial environmental risk (Dial & Vaughan, 1987; Nutting, 1969). This environmental risk during the mating flight could represent a high potential cost to inbreeding avoidance. This in turn could select for behavior that minimizes time spent aboveground, thus reducing the amount of mates that can be encounter, leading to higher inbreeding tolerance (Kokko & Ots, 2006). After the mating flight, the alate pair starts a colony that will undergo multiple reproductive episodes (Eggleton, 2010); thus, mate choice during this brief period can influence lifetime reproductive success across decades. Inbreeding between related alates regularly occurs in termite populations and can result in negative fitness effects (Calleri et al., 2006; DeHeer & Vargo, 2006), which makes it likely that inbreeding should preferentially be avoided if possible. Although sufficient dispersal distances can facilitate outbreeding (Husseneder et al., 2006; Vargo & Husseneder, 2010), termites are generally not considered as apt flyers (Nutting, 1969). Therefore, if inbreeding avoidance is important in this termite species, it is likely that kin recognition or differential sex ratios could also play a role. Alates of basal termite clades (lower termites) do not display kin recognition (but see Aguilera‐Olivares et al., 2015; DeHeer & Vargo, 2006; Husseneder & Simms, 2008; Kitade et al., 2004; Shellman‐Reeve, 2001), yet the capacity for kin recognition in derived termite lineages (higher termites) is poorly understood (Vargo & Husseneder, 2010). Differential bias in sex ratios between colonies has only been observed in lower termites (Husseneder et al., 2006). Thus, in higher termites, kin recognition and/or sex ratios could potentially play a vital role in reducing the risk of inbreeding when choosing a monogamous mate.

Organisms are predicted to equally allocate resources to male and female offspring (Fisher, 1930), and biased allocation can have positive or negative effects on offspring's mating and reproductive success. Sex allocation measurements, as opposed to sex ratio, not only incorporate the number of offspring, but also an energetic estimate of each sex. Generally, bias is maladaptive because it increases the reproductive value of the rarer sex or sex with lesser allocation (Fisher, 1930). However, adaptive bias may occur if a specific sex reaps higher fitness benefits under good environmental conditions, and thus receives greater allocation (Trivers & Hare, 1976). Bias can also improve mating success or resource procurement in cases of cooperation or competition with kin, such that allocation maximizes inclusive fitness (Hamilton, 1967; Clark,  1978; Taylor, 1981). In termites, adaptive sex allocation bias can further arise through within‐colony mother‐son or father‐daughter mating, which generates differential fitness between sexes (Hellemans et al., 2019; Kobayashi et al., 2013; Roisin & Lenz, 2002; Vargo et al., 2012). Conversely, maladaptive bias can be caused by sex allocation distorters like the bacterial symbiont *Wolbachia,* which commonly occurs in insects (Charlat et al., 2003; Correa & Ballard, 2016). *Wolbachia* is vertically transmitted by females, and thus the parasite can increase its fitness by inducing female bias through male‐killing, feminization, and parthenogenesis (Werren et al., 2008). Multiple termite taxa associate with *Wolbachia* (Bandi et al., 1997; Bordenstein & Rosengaus, 2005; Hellemans, Kaczmarek, et al., 2019; Lo & Evans, 2007; Roy & Harry, 2007; Salunke et al., 2010) but parasitic manipulation has yet to be observed.

Here, we evaluate inbreeding avoidance and sex allocation to infer the potential effects on termite mating and reproductive success. We utilized reproductive alates from the soil‐feeding higher termite *Cubitermes tenuiceps*, since moderate inbreeding and symbiosis with *Wolbachia* have been observed within the genus (Roy et al., 2010; Roy & Harry, 2007). Inbreeding avoidance was assessed based on kin recognition during a behavioral assay with a male choosing between a nestmate and non‐nestmate female from a local or foreign location; thus, determining if genetic or environmental cues affect mate choice. Differential sex ratio bias among colonies was examined by counting all alates from each colony prior to the nuptial flight. Sex allocation was then determined based on the number and dry weights of each sex, as dry weight is a common proxy used for energetic investment in termites and other social insects (Cremer & Heinze, 2002; Hellemans, Fournier, et al., 2019; Kobayashi et al., 2013; Sundstrom et al., 1996; Trivers & Hare, 1976). Finally, the incidence of *Wolbachia* infection in alates was examined to investigate whether this co‐occurred with sex allocation, indicating potential parasitism.

## 2 MATERIALS AND METHODS

### 2.1 Colony sampling and termite identification

Twelve nests of *Cubitermes tenuiceps* from two locations around Pretoria, South Africa, (Table S1) were systematically excavated in October 2019 when no rain had fallen for 5 months (South African Weather Service, weathersa.co.za), with the aim to collect all alates. Upon collection, alates were stored in boxes humidified with moist tissue paper, where they were kept for 1–2 hr before being brought to the laboratory at FABI, University of Pretoria. Alates were collected 1–2 days before the first rains, after which *Cubitermes* has its nuptial flight (Nutting, 1969). The day after the first rain, several colonies from both locations were excavated and no alates were found. Therefore, the alates collected should represent the full annual reproductive investment per colony.

### 2.2 Species identification

Soldiers from each colony were used for genus‐level classification (Uys, 2002) while DNA barcoding was used for species identification. Legs were removed from four males and four females from each colony for a DNA extraction using Chelex (0.2 ml 10% Chelex solution, 99.9°C for 15 min). We then amplified *Cytochrome c oxidase subunit II* (*COXII*) for each sample (Primers: *A‐tLeu* 5′‐CAGATAAGTGCATTGGATTT‐3′; (Miura et al., 1998); and *B‐tLys* 5′‐ GTTTAAGAGACCAGTACTTG‐3′; (Liu & Beckenbach, 1992). The PCR mix consisted of 12.5 µl Red Taq master mix (Ampliqon), 1.0 µl forward primer, 1.0 µl reverse primer, 0.1 µl bovine serum albumin (BSA), and 8.4 µl H_2_O for a total volume of 25 µl. PCR conditions were as follows: 94°C for 5 min, 35 cycles of 94°C for 10 s, 50°C for 20 s, and 72°C for 45 s. The program ended with a final elongation of 72°C for 7 min. PCR success was determined using agarose gel electrophoresis. PCR products from 2 specimens (1 female and 1 male) from each colony were then purified using MSB Spin PCRapace (STRATEC Molecular, Germany), and underwent Sanger sequencing at Eurofins (Ebersberg, Germany). The resulting sequences (1 for the forward and 1 for the reverse primer) for each specimen were then aligned using a pairwise *Geneious alignment* with default settings in Geneious 2019 (Biomatters Ltd., New Zealand). The highest‐quality consensus sequence from each colony was checked against the NCBI nucleotide database using BLASTn.

### 2.3 Inbreeding avoidance: kin recognition during mate choice and sex ratios

**FIGURE 1:**
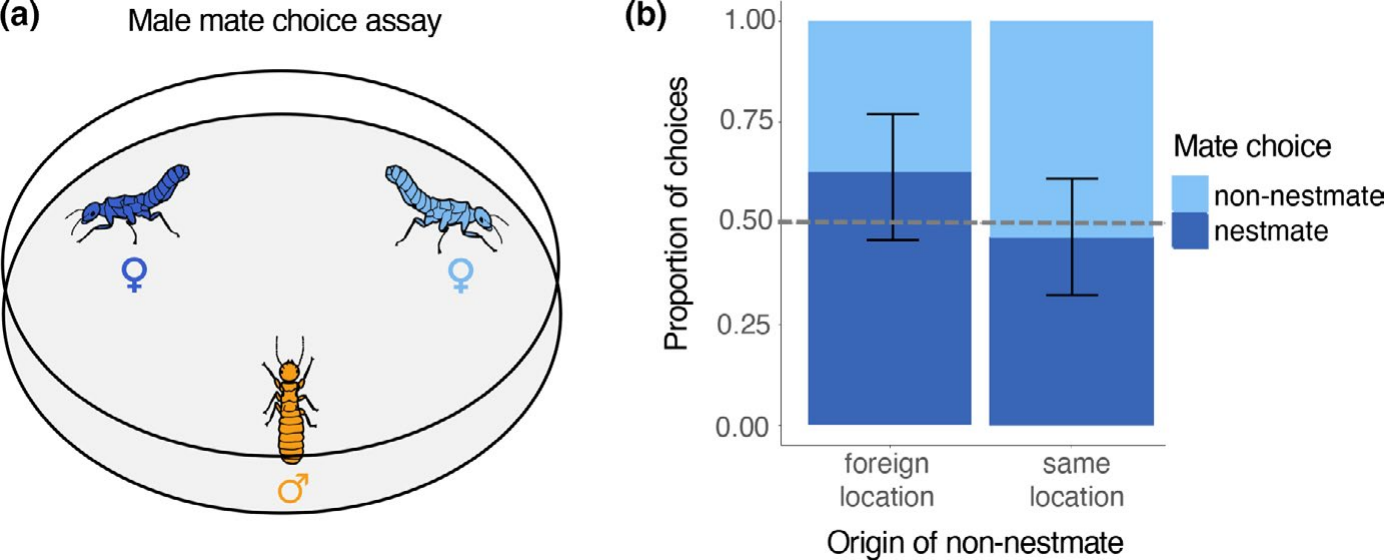
(a) Schematic of mate choice test between nestmate and non‐nestmate females, whose raised abdomens demonstrate calling behavior to attract a male. (b) Proportion of males that preferred nestmate or non‐nestmate females (choice‐tests: *n*_foreign_ = 35; *n*_same_ = 43). Male choice did not significantly differ from random, which is shown by the dotted line, regardless of non‐nestmate female location of origin. Error bars indicate 95% confidence intervals

In *Cubitermes*, similar to many termites, initial mate choice during pairing is performed by males. Female *Cubitermes* raise their abdomen in calling behavior, presumably releasing sex‐pairing pheromones to attract a male (Bordereau & Pasteels, 2010; Williams, 1959). Subsequently, males choose and follow the female in a tandem run until they establish a nest. Males occasionally exhibit choosiness by changing female partner during the tandem run (Williams, 1959). Therefore, we examine male mate choice between females during pairing and tandem running. Mate choice assays were conducted by observers who did not know the origin of the female. The observers monitored male choice and a coordinator that recorded the colony of origin of the termites. Two females, a nestmate and a non‐nestmate from either the same or foreign collection location, were placed away from each other, in an arena (90 mm Ø Petri dish) with soil from the local environment. The observer followed one of the females, assigned as the focal female, for the remainder of the assay. The females were given 1 min to acclimatize, during which they would often raise their abdomens. Sometimes females would walk around for some time before standing still. In the rare occasion that females met each other, they appeared to ignore each other and continue walking until raising their abdomen. After acclimatizing, a male was added (Figure 1a). After 3 min, his choice of female (focal, nonfocal, or no choice) was communicated to the coordinator. The coordinator marked whether the chosen female was nestmate or non‐nestmate. During the observation period, few males changed their choice of female, and only the final choice was recorded.

Termites were only used for a single assay, resulting in 78 assays of mate choice (*n* = 35 non‐nestmate from foreign location, *n* = 43 non‐nestmate from same location), in which eleven colonies were used (Table S2). The data were analyzed using a binomial generalized linear mixed model (GLMM) with “mate choice” as the dependent variable, “female origin” the predictor, and “male colony of origin” a random effect. “No choice” occurred 14 times and was not considered in the data analysis. To test whether mate choice was random, we implemented an intercept offset using the logit of 0.5.

Heterogeneity between colonies was tested using a *G*‐test.

Sex ratios were determined by quantifying the number of male and female alates found in each colony (*n*_colony_ = 11; Table S2). Alates were sexed using morphological characteristics (Krishna et al., 2013), and sex ratio was calculated by dividing the number of males by the total number of alates. For each colony, we analyzed whether sex ratio deviated from 0.5, representative of a balanced sex ratio, using a test of equal proportions (prop.test function, R).

### 2.4 Sex allocation and *Wolbachia* identification

In addition to the examination of sex ratios, energy allocation to each sex was estimated by measuring the dry weight of ten males and ten females per colony (*n*_colony_ = 9; Table S2). Termites were euthanized at −20°C, dried at 60°C for 48 hr, and then weighed. Data were analyzed using a LMM in the *lme4* package (Bates et al., 2015, p. 4) in *R* (R Core Team, 2018) with “dry weight” as the dependent variable, “sex” as the predictor and “colony” as a random effect. Additionally, the total investment in each sex was estimated by multiplying the number of alates of a sex by their average dry weight per colony. The estimated colonial energetic allocation was then analyzed using a linear mixed‐effects model (LMM; *lmer* function, *lme4* package), with the logarithm of “total weight” as the dependent variable, “sex” as the predictor, and “colony” as a random effect. In addition, a 0.7 power conversion factor was applied to total female‐to‐male dry weight ratio to correct potential disparities between metabolic rate and dry weight proxies for energetic allocation, which has been found in ants (Boomsma, 1989; Boomsma et al., 1995). This conversion factor in ants is driven partly by differences in biology and partly by differences in size, and thus might not be appropriate for termites. However, studies in termites are lacking, and thus we include both analyses with and without conversion factor.

We screened for the presence or absence of *Wolbachia* to test whether differences in sex allocation co‐occurred with the symbiont. Using the DNA extracts from the termite species identification, we ran PCRs with *Wolbachia*‐specific primers for two different regions: 16S rRNA (16SWol‐F: 5′‐TTGTAGCCTGCTATGGTATAACT‐3′ and 16SWol‐R: 5′‐GAATAGGTATGATTTTCATGT‐3′; (O'Neill et al., 1992)) and the *ftsZ* gene (ftsZuniF: 5′‐GGYAARGGTGCRGCAGAAGA‐3′ and ftsZuniR: 5′‐ATCRATRCCAGTTGCAAG‐3′; (Lo et al., 2002)). The PCR mix was the same as for *COXII* and had a total volume of 25 µl. The conditions for the PCR followed Lo et al. (2002). Successful amplification was confirmed using agarose gel electrophoresis. To confirm that it was *Wolbachia* which had been successfully amplified, a subset of *ftsZ* PCR products were purified using MSB Spin PCRapace (STRATEC Molecular, Germany), and underwent Sanger sequencing at Eurofins (Ebersberg, Germany). The resulting forward and reverse sequences for each primer were aligned using a pairwise *Geneious alignment* with default settings in Geneious 2019 (Biomatters Ltd., New Zealand), and the consensus sequences were checked against the NCBI nucleotide database using BLASTn.

## 3 RESULTS

### 3.1 Termite identification

All colonies except one were identified to *Cubitermes tenuiceps*, with 97%–98% nucleotide sequence similarity to the BLASTn hit MN685946.1 in GenBank. The remaining colony also matched best *Cubitermes tenuiceps*, but with a lower match of 92%, so we removed it from further analyses.

### 3.2 Inbreeding avoidance: no apparent kin recognition during mate choice but female‐biased sex ratios

**FIGURE 2:**
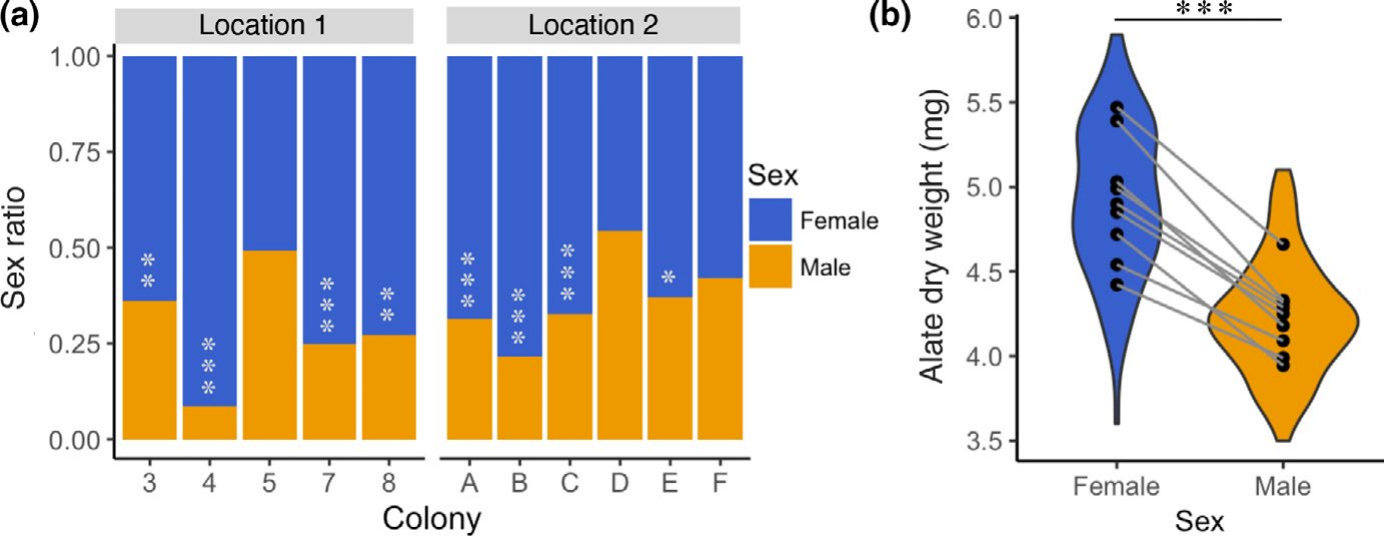
(a) Sex ratio denoted by proportion of male and female alates for each colony from both locations. Asterisks indicate significant deviation from 0.50 (*p < .05, **p < .01, ***p < .001). (b) Female alates exhibit a higher dry weight than their male counterparts. Each dot represents the average of a colony and the violin shape illustrates the distribution of individual weights. Lines connect males and females from the same colony

Our observations matched previously published literature in that only males appear to be involved in mate choice. As soon as a male taps a female on the abdomen with his antennae, or attaches to the female abdomen with his mandibles, the female starts running and finding a place to dig a nest. The female never appears to turn around to contact the male, and the male continuously remains in contact with the female's abdomen during this process. Overall, mate choice appeared random (Figure 1, GLMM, intercept, *z* = 1.60, *p* = .11). When males had a choice between a nestmate female and a non‐nestmate female from the same location, males chose a nestmate female during 45.5% of the trials. When the non‐nestmate female was from a different location, males joined a nestmate female in 62.9% of the trials. This difference in choice depending on location was, however, not significant (Figure 1; GLMM: location, *z* = −1.42, *p* = .16). Colonies were homogeneous in their choice of mate (*G*‐test, *G* = 12.618, *df* = 10, *p* = .25, Figure S1). The sex ratios of colonies were largely female‐biased. The average sex ratio was 0.33 ± 0.13 (mean ± *SD*) and significantly female‐biased in 8 out of 11 colonies (Figure 2a, Table S4). No colonies exhibited male‐bias.

### 3.3 Female‐biased sex allocation and ubiquitous presence of *Wolbachia*

Sex allocation was also found to be largely female‐biased. Dry weights differed significantly between sexes (Figure 2b; LMM, Sex, χ^2^ = 213.73, *p* < .001, Table S5), with females (4.92 ± 0.35 mg, mean ± *SD*) being heavier than males (4.23 ± 0.21 mg). Thus, sex significantly affected the total energetic investment (LMM, Sex, χ^2^ = 30.80, *p* < .001), represented by the estimated cumulative dry weight of each sex per colony (Figure S2). The ratio of female‐to‐male dry weight remained female‐biased with the power conversion factor (Table S6), further corroborating our findings of skewed sex allocation.

*Wolbachia* was successfully amplified for both regions for alates from all colonies (Table S2). All sequenced PCR products were closest to *Wolbachia* from the termite *Kalotermes flavicollis* (98.4%–98.8% sequence similarity; GenBank: AJ292345), with the exception of *Wolbachia* from colony B, which matched closest to *Wolbachia* from a bush cricket (Bit score: 1,229; sequence identity: 96.9; GenBank: DQ536100) but had highest sequence identity to the *K. flavicollis Wolbachia* (Bit Score: 1,227; sequence identity: 97.0%). *Wolbachia* from both of the closest BLAST hits are within the *Wolbachia* group F (Lo & Evans, 2007; Panaram & Marshall, 2007), which is commonly associated with termites (Lo & Evans, 2007).

## 4 DISCUSSION

We sought to investigate characteristics of inbreeding avoidance and sex allocation, which may influence the mating and reproductive success of the higher termite *Cubitermes tenuiceps*. No apparent male mate choice was found, and sex ratios were female‐biased. This suggests that *C. tenuiceps* neither avoids inbreeding through kin recognition nor through differential sex ratios. As females were heavier than males, sex allocation was also female‐biased. *Wolbachia* was consistently present in alates, warranting further research into potential parasite distortion of termite sex allocation.

Although the cost of inbreeding has not been quantified in *C. tenuiceps*, there is evidence from other termites that costs can be high (DeHeer & Vargo, 2006), for example in regards to compromised immune defenses (Calleri et al., 2006). If inbreeding has a tangible fitness cost in this monogamous species, reproductive alates must either rarely encounter related individuals, for example, due to sufficient dispersal during mating flights (Vargo & Husseneder, 2010), or trade‐offs / evolutionary constraints must exist that prevent the evolution of inbreeding avoidance. Mating flights are extremely dangerous (Nutting, 1969), which could lead to males choosing the first female they encounter regardless of relatedness (Waser et al., 1986). Another potential trade‐off may be related to pathogen pressure. In the termites *Zootermopsis angusticollis* and *Coptotermes formosanus*, mating with an unrelated individual increases mortality of the mated pair, likely due to exposure to foreign fungi and bacteria (Fei & Henderson, 2003; Rosengaus & Traniello, 1993). Increased risk of infection could outweigh any inbreeding costs. Our results show a trend, although nonsignificant, that males prefer nestmate females when the non‐nestmate female is from a different location. If different locations harbor different pathogens, inbreeding could be adaptive to avoid novel pathogens.

Differentially biased sex ratios, where colonies specialize in producing either males or females to prevent sib‐mating, can effectively reduce the effect of inbreeding (Noirot, 1989) and have been observed in *Coptotermes formosanus* (Husseneder et al., 2006). However, we found female‐biased sex ratios for all but three colonies, which did not demonstrate male‐bias, indicating that sex ratio biases would not counter inbreeding. The deviation from equal investment in the sexes suggests other traits potentially drive female bias and impact fitness.

The drivers and fitness consequences of the female‐biased sex allocation remain unclear, although certain adaptive cases are unlikely due to termite biology. Local mate competition, resource competition, or resource enhancement can promote sex allocation bias to improve mating success or resource procurement of offspring or kin (Hamilton, 1967; Clark,  1978; Taylor, 1981). Mate competition between related males can cause female bias in order to enhance male reproductive success through multiple mating (Hamilton, 1967; Taylor, 1981). However, monogamy, as exhibited by most termites, nullifies any benefit of multiple mating and reduces mate competition, making the optimal sex allocation equally favor males and females (West et al., 2000). Additionally, alates of higher termites rarely remain in their natal nest or interact during nest‐founding, reducing the potential for resource enhancement via cooperation among kin (but see Eggleton, 2010; Roisin, 2000), and *Cubitermes*' food source (soil) (Uys, 2002), is a ubiquitous resource, likely minimizing resource competition.

Two other traits predicted to affect investment, within‐colony mating (Kobayashi et al., 2013; Roisin & Lenz, 2002) and environmental conditions (Trivers & Willard, 1973), could potentially cause the sex allocation bias to benefit the termites. Within‐colony mating with son or daughter reproductive replacements can beneficially bias sex allocation to favor the parent with greater genetic contribution (Kobayashi et al., 2013; Roisin, 2000; Roisin & Lenz, 2002). However, it is unlikely that within‐colony mating promotes the female bias in *C. tenuiceps*. Reproductive replacements normally occur in a smaller fraction of termite colonies than observed in this study (Hellemans, Fournier, et al., 2019; Kobayashi et al., 2013; Vargo et al., 2012), and replacements are thought to be uncommon in *Cubitermes* (Myles, 1999; Noirot, 1956). Alternatively, sex allocation may be biased if fitness of the sexes varies with environmental quality, such that the sex that reaps higher fitness benefits from a better environment should receive greater allocation in good conditions (Kümmerli & Keller, 2011; Trivers & Willard, 1973). Thus, termites could theoretically invest more in females if they had greater fitness under favorable environmental conditions, reinforcing the production of this energetically costly sex. However, conditional sex allocation related to environmental quality remains to be demonstrated in termites. Overall, the lack of evidence for within‐colony mating or advantageous environmental conditions prevents any firm conclusions of adaptive sex allocation.

The female‐biased sex allocation may be maladaptive and potentially relates to the ubiquitous prevalence of *Wolbachia*. The inflated number of females during the nuptial flight may reduce the mating success and increase the reproductive value of the rarer male sex (Fisher, 1930). Thus, biased allocation toward females potentially reduces fitness, and the frequency‐dependent nature of selection should cause colonies to invest more in males, unless a parasitic sex distorter is driving the female bias (Fisher, 1930; West, 2009). *Wolbachia* is well‐known female‐biased sex allocation distorter in arthropods (Werren et al., 2008). This bacterial symbiont is vertically transmitted through females, and the symbiont can maximize transmission through reproductive parasitism (Miller & Schneider, 2012; Werren et al., 2008) in the form of feminization of males, male‐killing, and/or parthenogenesis induction (Charlat et al., 2003; Correa & Ballard, 2016). All alates surveyed were infected with *Wolbachia*, similar to the high incidence in alates of *Cubitermes* sp. *affinis subarquatus* (Roy et al., 2015), and other termites across derived (higher termites) and basal (lower termites) clades also host *Wolbachia* (Bandi et al., 1997; Bordenstein & Rosengaus, 2005; Hellemans, Kaczmarek, et al., 2019; Lo & Evans, 2007; Roy & Harry, 2007; Salunke et al., 2010). However, the exact nature of the symbiosis in termites remains largely unknown (but see Hellemans, Kaczmarek, et al., 2019), and a parasitic association cannot be assumed without causal proof. For example, variable sex allocation, which is driven by parthenogenesis and within‐colony inbreeding, and *Wolbachia* co‐occur in some other termites without being causally linked (Hellemans, Fournier, et al., 2019; Matsuura et al., 2004; Yashiro & Lo, 2019). If *Wolbachia* caused the observed female‐biased sex allocation, this may generate fitness costs due to reduced mating success during the nuptial flight and diminished reproductive success due to male‐killing of offspring (Charlat et al., 2003). Thus, the symbiotic nature of *Wolbachia* in *Cubitermes* is deserving of further research, particularly if it acts as a reproductive parasite and consequentially reduces host fitness.

## CONFLICT OF INTEREST

The authors declare no conflicts of interest.

## AUTHOR CONTRIBUTIONS

**Veronica M. Sinotte:** Investigation (equal); methodology (equal); resources (equal); visualization (equal); writing‐original draft (equal); writing‐review & editing (equal). **Benjamin H. Conlon:** Conceptualization (equal); data curation (equal); formal analysis (equal); investigation (equal); methodology (equal); writing‐original draft (equal); writing‐review & editing (equal). **Elena Seibel:** Investigation (equal); methodology (equal); writing‐review & editing (equal). **Jan W. Schwitalla:** Investigation (equal); methodology (equal); writing‐review & editing (equal). **Z. Wilhelm de Beer:** Resources (equal); writing‐review & editing (equal). **Michael Poulsen:** Resources (equal); writing‐review & editing (equal). **Nick Bos:** Conceptualization (lead); formal analysis (lead); investigation (equal); methodology (equal); visualization (equal); writing‐original draft (equal); writing‐review & editing (equal).

## Supporting information

Supplementary Material

## Data Availability

Sequences generated in this study were deposited to GenBank (Table S3). Data on mate choice and sex ratios have been uploaded to datadryad: (https://datadryad.org/stash/share/35OzGoQ1YovGwOmqtD2gbXVMl4YzilocKQc1_srY0YI).
