# Supplementary material for: Female‐biased sex allocation and lack of inbreeding avoidance in Cubitermes termites
Source: Ecol Evol. 2021 Mar 31;11(10):5598–605. doi: 10.1002/ece3.7462 (PMC8131773; doi:10.1002/ece3.7462)
Supplement: Supplementary file 1 — Supplementary Material [file ECE3-11-5598-s001.docx]

**Female-biased sex allocation and lack of inbreeding avoidance in *Cubitermes* termites**

**Electronic Supplementary materials**

**Supplementary tables and figures**

**Table S1.** Coordinates of colonies from field site 1 and 2, which were located 3.2 km apart. The colonies were collected in Pretoria, South Africa from the field belonging to the Agricultural Research Council (ARC) or the University of Pretoria’s Experimental Farm (EF).

| Colony ID | Field site | Location | GPS coordinates |
| --- | --- | --- | --- |
| 3 | 1 | ARC | -25.7425590232015, -25.7425590232015 |
| 4 | 1 | ARC | -25.7426600251347, 28.2615319825709 |
| 5 | 1 | ARC | -25.7426890265197, 28.2614380214363 |
| 6 | 1 | ARC | -25.7417869661003, 28.2614240236580 |
| 7 | 1 | ARC | -25.7421120163053, 28.2612810283899 |
| 8 | 1 | ARC | -25.7420830149204, 28.2610859815031 |
| A | 2 | EF | -25.7297089789063, 28.2327190227807 |
| B | 2 | EF | -25.7297280058265, 28.2324969861656 |
| C | 2 | EF | -25.7298180274665, 28.2323389872909 |
| D | 2 | EF | -25.7302829716355, 28.2403759751469 |
| E | 2 | EF | -25.7304139807820, 28.2405980117619 |
| F | 2 | EF | -25.7304030004889, 28.2406389992684 |

**Table S2.** The experiments within the study and the respective colonies from which alates were used. For the last column, X indicates one individual, XX indicates two individuals that were barcoded (one from each sex).

| Colony ID | Sex ratios | Dry weight | Mate choice | Colony barcoded | *Wolbachia* amplified | *Wolbachia* barcoded |
| --- | --- | --- | --- | --- | --- | --- |
| 3 | X | X | X | X | X | X |
| 4 | X |  | X | X | X (no male) | X |
| 5 | X |  | X | X | X | XX |
| 6 |  |  |  | X | X |  |
| 7 | X | X | X | X | X (no male) | XX |
| 8 | X | X | X | X | X | XX |
| A | X | X | X | X | X (no male) |  |
| B | X | X | X | X | X | XX |
| C | X | X | X | X | X | X |
| D | X | X | X | X | X |  |
| E | X | X | X | X | X |  |
| F | X | X | X | X | X | X |

**Table S3.** The colony of origin used for sequences *COXII* and ftsZ to identify *Cubitermes* and *Wolbachia*, respectively, and the corresponding accession numbers in GenBank.

| Genome | Gene | Colony ID | GenBank ID |
| --- | --- | --- | --- |
| mitochondrial | *COXII* | 3 | MT702620 |
| mitochondrial | *COXII* | 4 | MT702621 |
| mitochondrial | *COXII* | 5 | MT702622 |
| mitochondrial | *COXII* | 6 | MT702623 |
| mitochondrial | *COXII* | 7 | MT702624 |
| mitochondrial | *COXII* | 8 | MT702625 |
| mitochondrial | *COXII* | A | MT702626 |
| mitochondrial | *COXII* | B | MT702627 |
| mitochondrial | *COXII* | C | MT702628 |
| mitochondrial | *COXII* | D | MT702629 |
| mitochondrial | *COXII* | E | MT702630 |
| mitochondrial | *COXII* | F | MT702631 |
| *Wolbachia* | *ftsZ* | 4 | MT754250 |
| *Wolbachia* | *ftsZ* | 5 | MT754251 |
| *Wolbachia* | *ftsZ* | 7 | MT754252 |
| *Wolbachia* | *ftsZ* | 8 | MT754253 |
| *Wolbachia* | *ftsZ* | B | MT754254 |
| *Wolbachia* | *ftsZ* | 7 | MT754255 |
| *Wolbachia* | *ftsZ* | 8 | MT754256 |
| *Wolbachia* | *ftsZ* | 5 | MT754257 |
| *Wolbachia* | *ftsZ* | C | MT754258 |
| *Wolbachia* | *ftsZ* | 3 | MT754259 |
| *Wolbachia* | *ftsZ* | B | MT754260 |
| *Wolbachia* | *ftsZ* | F | MT754261 |

**Table S4.** Test of equal proportions in colony sex ratios. n = the number of individuals included per colony.

| Colony ID | n | *X^2^* | df | P |
| --- | --- | --- | --- | --- |
| 3 | 130 | 9.423 | 1 | 0.0021 |
| 4 | 58 | 38.09 | 1 | 6.77E-10 |
| 5 | 106 | 0.009 | 1 | 0.9226 |
| 7 | 89 | 21.75 | 1 | 3.10E-06 |
| 8 | 55 | 10.47 | 1 | 0.0012 |
| A | 96 | 12.76 | 1 | 0.0004 |
| B | 83 | 25.49 | 1 | 4.44E-07 |
| C | 159 | 18.34 | 1 | 1.85E-05 |
| D | 171 | 1.146 | 1 | 0.2843 |
| E | 81 | 4.938 | 1 | 0.0263 |
| F | 69 | 1.449 | 1 | 0.2286 |

**Table S5.** Results of a linear model per colony, with dry weight of each measured individual as the dependent variable, and sex as the predictor.

| Colony ID | Estimate | t | P |
| --- | --- | --- | --- |
| 3 | -0.4322 | -2.316 | 0.0333 |
| 7 | -0.45 | -4.017 | 0.0008 |
| 8 | -1.06 | -7.354 | 7.96E-07 |
| A | -0.7756 | -5.928 | 1.65E-05 |
| B | -0.6733 | -3.418 | 0.0042 |
| C | -0.85 | -7.630 | 4.78E-07 |
| D | -0.6111 | -4.588 | 0.0003 |
| E | -0.81 | -6.577 | 3.53E-06 |
| F | -0.6 | -5.051 | 8.32E-05 |

**Table S6.** Female-to-male dry weight cost ratio and power correction. The total dry weight ratio was calculated by multiplying the number of alates from each sex by the average dry weight for that colony. The correct individual and total dry weight ratios were performed with a 0.7 power conversion factor.

| Colony ID | Individual dry weight ratio | Corrected individual dry weight ratio | Total dry weight ratio | Corrected total dry weight ratio |
| --- | --- | --- | --- | --- |
| 3 | 1.766 | 1.489 | 1.952 | 1.597 |
| 7 | 3.045 | 2.181 | 3.378 | 2.344 |
| 8 | 2.667 | 2.667 | 2.667 | 2.667 |
| A | 2.200 | 1.737 | 2.644 | 1.975 |
| B | 3.611 | 2.457 | 4.154 | 2.710 |
| C | 2.058 | 1.657 | 2.479 | 1.888 |
| D | 0.839 | 0.884 | 0.957 | 0.970 |
| E | 1.700 | 1.450 | 1.993 | 1.620 |
| F | 1.379 | 1.252 | 1.577 | 1.376 |


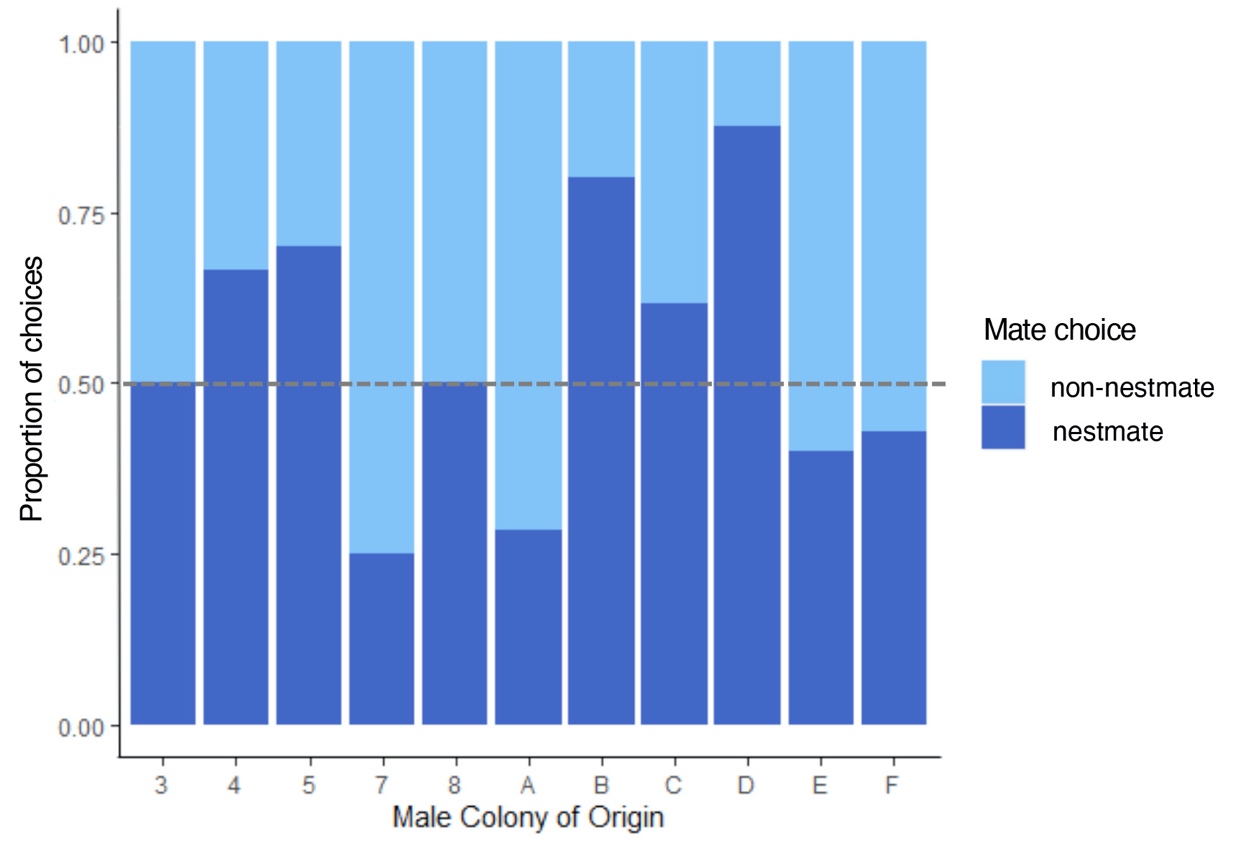


**Figure S1.** Proportion of males that chose non-nestmate or nestmate females, visualized based on male colony of origin.

**
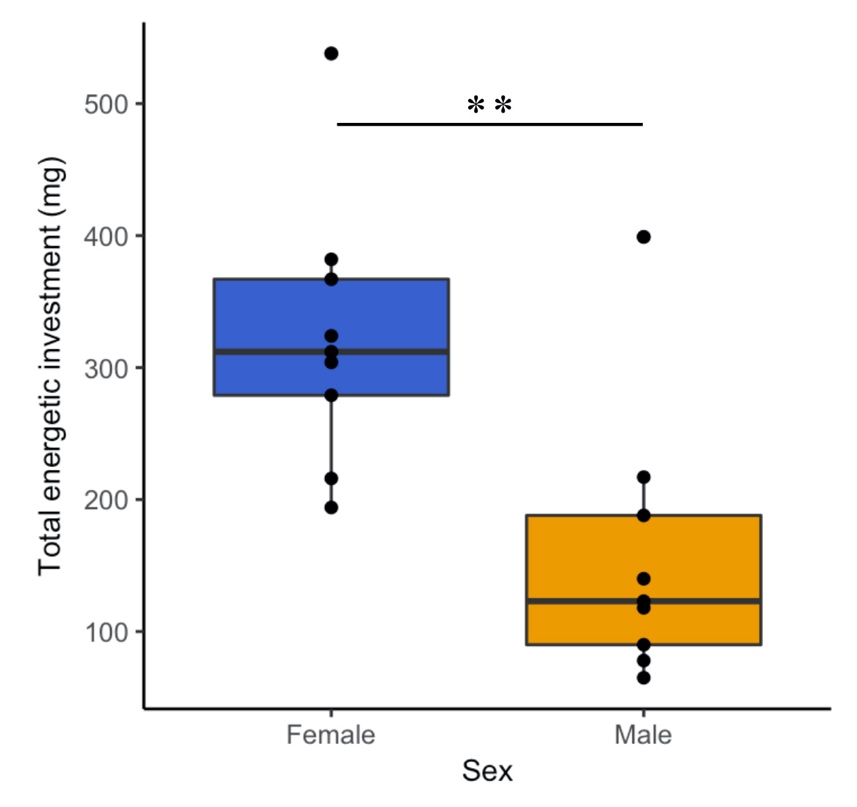
**

**Figure S2.** Total energetic investment of colonies in male and female alates. The energetic investment is based on the estimated total dry weight of each sex from colonies. Colonies invest significantly more in females than males (P<0.001), demonstrating female-biased sex allocation.
